# Lipids of Archaeal Viruses

**DOI:** 10.1155/2012/384919

**Published:** 2012-09-20

**Authors:** Elina Roine, Dennis H. Bamford

**Affiliations:** Department of Biosciences and Institute of Biotechnology, University of Helsinki, P.O. Box 56, Viikinkaari 5, 00014 Helsinki, Finland

## Abstract

Archaeal viruses represent one of the least known territory of the viral universe and even less is known about their lipids. Based on the current knowledge, however, it seems that, as in other viruses, archaeal viral lipids are mostly incorporated into membranes that reside either as outer envelopes or membranes inside an icosahedral capsid. Mechanisms for the membrane acquisition seem to be similar to those of viruses infecting other host organisms. There are indications that also some proteins of archaeal viruses are lipid modified. Further studies on the characterization of lipids in archaeal viruses as well as on their role in virion assembly and infectivity require not only highly purified viral material but also, for example, constant evaluation of the adaptability of emerging technologies for their analysis. Biological membranes contain proteins and membranes of archaeal viruses are not an exception. Archaeal viruses as relatively simple systems can be used as excellent tools for studying the lipid protein interactions in archaeal membranes.

## 1. Introduction

Viruses are obligate parasites. Their hallmark is the virion, an infectious particle made of proteins and encapsidating the viral genome. Many viruses, however, also contain lipids as essential components of the virion [1]. The majority of viral lipids are found in membranes, but viral proteins can also be modified with lipids [2, 3]. 

### 1.1. Membrane Containing Viruses in the Viral Universe

**Figure 1:**
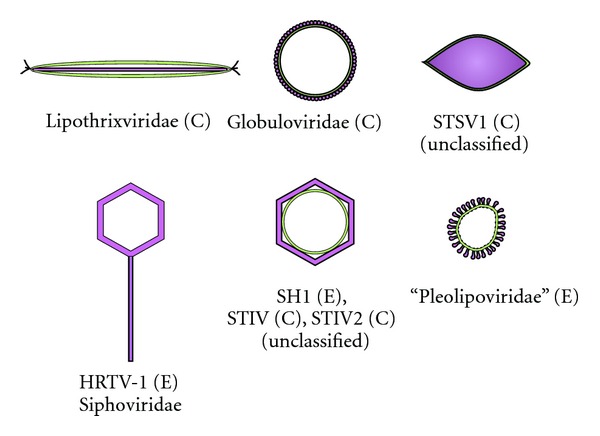
Schematic representation of the currently known lipid containing archaeal viruses (C = viruses infecting crenarchaeal hosts, E = viruses infecting euryarchaeal hosts). As a comparison, an archaeal virus devoid of a membrane [32] is also shown. Membrane is illustrated as a yellow layer either inside or outside of the protein capsid depicted in purple. The viral particles are not drawn in scale.

Membrane containing viruses can roughly be divided into two subclasses [1]. The first subclass contains viruses in which the membrane, also called an envelope, is the outermost layer of the viral particle. In the second class of viruses, the membrane is underneath the usually icosahedral protein capsid. Few viruses contain both the inner membrane as well as an envelope [1]. Lipid membranes of viruses have evolved into essential components of virions that in many cases seem to be involved in the initial stages of infection [4–6]. The majority of membrane containing viruses infect animals both vertebrate and invertebrate that do not have a cell wall surrounding the cytoplasmic membrane. For other host organisms such as plants and prokaryotes there are much fewer membrane containing viruses known [1]. Usually the cells of these organisms are covered with a cell wall. By far the majority of known viruses that infect prokaryotes, that is, bacteria (bacteriophages), and archaea (archaeal viruses) belong to the order *Caudovirales*, the tailed viruses (Figure 1) [1, 7]. These viruses are made of the icosahedrally organized head and a helical tail. Tailed viruses do not usually contain a membrane, although there are some early reports of tailed mycobacteriophages containing lipids [8, 9]. Viral proteins can also be modified with lipids [3], and there are some indications that proteins of archaeal viruses may also contain lipid modifications [10]. Since very little is known about the lipid modifications of archaeal virus proteins, this paper will concentrate mostly on the membrane lipids of archaeal viruses. 

### 1.2. How Do Viruses Obtain Membranes?

Viral-encoded genes possibly involved in lipid modifications have been found in large eukaryotic viruses such as Mimivirus [11] and *Paramecium bursaria *Chlorella virus 1 (PBCV-1) [12]. In prokaryotic viruses, however, no genes encoding components for lipid metabolism have been recognized, but the membranes of prokaryotic viruses are mostly obtained from the host cytoplasmic membranes [13, 14]. Enveloped viruses obtain the membrane during budding, that is, egress of the viral particles from the cells without disturbing the cell membrane integrity [15]. The inner membrane of prokaryotic viruses is presumed to be obtained from specific patches of host cytoplasmic membrane containing viral membrane proteins and mechanistically analogous to the formation of clathrin coated pits [6, 16–18]. Consequently, enveloped viruses often exit the cells without lysis, whereas the viruses containing a membrane inside the capsid usually lyse the cells. At least one exception to this can be noted. Prokaryotic lipid containing virus *ϕ*6 contains an envelope, but its infection cycle ends in lysis of the host cells [19, 20]. 

As mentioned above, viral membranes are often involved in the initial stages of infection. This is especially true for the enveloped viruses where the proteins responsible for host recognition (spikes or fusion proteins) are usually incorporated in the envelope. At some point during the often multiphase entry process, the viral envelope fuses with a host membrane releasing the contents into the cell [4, 5]. Among viruses that contain the membrane inside the capsid, the involvement of the membrane in the entry has been shown for the bacteriophage PRD1. After the receptor recognition, the protein rich membrane forms a tubular structure through which the DNA enters the cell cytoplasm [21–23]. Such tubular structures, however, are not formed by all prokaryotic icosahedral viruses containing the membrane inside the capsid [24–28]. For the bacteriophage PM2 that infects the marine bacterium *Pseudoalteromonas*, fusion of the viral inner membrane with the host outer membrane was suggested [29]. Similarly, fusion of the *Sulfolobus* turreted icosahedral virus (STIV) membrane with the cytoplasmic membrane of *S. solfataricus *was suggested [30]. In addition to the function in viral entry, the inner membrane of viruses act, together with the viral membrane proteins, as the scaffold for capsid protein assembly [18, 26, 31].

## 2. Analysis of Viral Membranes

How do we know if a membrane is part of the viral structure? Chloroform treatment can be used as the first step in screening for viral membranes: the infectivity of the virus is usually considerably reduced if the virions contain a membrane [32–34]. Chloroform treatment can, however, also abolish the infectivity of virions that have not been reported to contain lipids [35] and therefore further studies are always required. Low buoyant density is also an indicator of the lipid membrane in the virions [6, 36]. Sudan Black B can be used to stain the polyacrylamide gel containing separated virion proteins and lipids [10, 37]. Although Sudan Black B is not entirely specific for lipids, positive staining is an indication of the presence of lipid membranes in highly purified viral material and also shows if some viral proteins are putatively lipid modified [10]. Further proof for the presence of a lipid membrane and analyses of its different components can be obtained by techniques also used for the analyses of the membrane lipids of the host cells, for example, thin layer chromatography (TLC), mass spectrometry (e.g., electrospray ionization, ESI-MS), and nuclear magnetic resonance (NMR) [38, 39]. Lipids must be obtained from highly purified viral material [10, 40, 41] or from distinct dissociation components of the virion [28, 42] as it is often difficult to separate virions from membrane vesicles of host origin.

## 3. Archaeal Lipids

Since the membrane lipids of archaeal viruses are derived from the host lipid pool, analysis of the host lipids is an important part of the lipid analysis of their viruses. Archaeal lipids are known to be drastically different from the ones of bacterial and eukaryotic membranes: instead of lipids based on diacylglycerol the most common core lipid of archaeal phospholipids is the diether of diphytanylglycerol [43, 44]. Archaeal lipids can be divided according to the two major kingdoms of Archaea. As a crude generalization, one can say that the haloarchaeal cell membranes consist mostly of bilayer-forming diether lipids, whereas membranes of archaeal thermophilic organisms are largely composed of tetraether lipids that form monolayer membranes [38, 45, 46]. As in other organisms, phospholipids are the major components of archaeal membranes. In halophilic Archaea, approximately 10% of total lipids are neutral lipids such as bacterioruberin [38]. The major core structure of haloarchaeal lipids consists of archaeol, a 2,3-di-*O*-phytanyl-*sn-*glycerol with C_20_ isoprenoid chains [38, 43]. One of the major lipids in extremely thermophilic archaea such as *Sulfolobus *sp. is the macrocyclic tetraether lipid caldarchaeol [46–48]. The composition of lipid membranes is modified according to the environmental conditions in all organisms, and Archaea are not an exception [38, 45, 47, 49].

Some archaeal proteins are known to be modified by isoprenoid derivatives [2, 60–62], and structural analysis revealed a diphytanylglyceryl methyl thioether lipid of one modified protein [61]. Modification of the *Haloferax volcanii *S-layer protein with a lipid of unknown structure was shown to be crucial to the maturation of the protein [62]. 

## 4. Archaeal Membrane Containing Viruses 

Our knowledge of archaeal viruses is scarce, but even less is known about their lipids. The known archaeal membrane containing viruses are listed in Table 1. Especially crenarchaeal viruses that infect thermophilic or hyperthermophilic hosts are difficult to produce in amounts high enough for closer analysis of their membrane lipids by traditional methods (e.g., [58]). 

### 4.1. Crenarchaeal Viruses

The presence of lipid membranes have been reported for icosahedral crenarchaeal viruses STIV and *Sulfolobus *turreted icosahedral virus 2 (STIV2), for filamentous viruses *Acidianus *filamentous virus 1 (AFV-1), *Sulfolobus islandicus *filamentous virus (SIFV), and *Thermoproteus tenax* virus 1 (TTV1), for spindle-shaped *Sulfolobus tengchongensis *spindle-shaped virus 1 (STSV1), and for spherical virus *Pyrobaculum *spherical virus (PSV; Table 1). The *Acidianus *bottle-shaped virus (ABV) virions were reported to contain a 9 nm thick envelope [63]. The lipid nature of this envelope, however, has not been reported and the estimated thickness of 9 nm is more than that of usual membranes of archaea [28, 58, 64, 65]. 

The case of spindle-shaped viruses such as *Sulfolobus *spindle-shaped virus 1 (SSV1) of family *Fuselloviridae* is interesting, because the virions have a buoyant density that is in the same range as in those virions containing a membrane (1.24 g/cm^3^ in CsCl), and the virions are sensitive to chloroform [66]. It has been reported that “10% of the SSV-1 virion envelope consists of host lipids” [67], but no further membrane studies have been conducted. This all may suggest that some other type of lipid component than a lipid membrane is present. 

The situation among the members of the family *Lipothrixviridae *is also confusing, because these viruses are defined as rod-shaped viruses containing an envelope. The family is further divided into genera *Alpha-*,* Beta-*,* Gamma-*, and *Deltalipothrixvirus* according to the specific structures involved in the host attachment located in the virion ends [68]. The envelope is reported to consist of viral proteins and host derived lipids [68]. The presence of a lipid envelope has been shown for alphalipothrixvirus TTV1 [51], betalipothrixvirus SIFV, and gammalipothrixvirus AFV-1 [53]. However, no evidence for a lipid membrane in the type species of *Deltalipothrixvirus *genus, the *Acidianus *filamentous virus 2 (AFV-2), could be found [69].

Further analysis using thin layer chromatography (TLC) has been reported for AFV1 [53], SIFV [54], STSV1 [59], and PSV [50]. The lipid composition of STIV was analysed using ESI-MS [70]. In conclusion, it could be shown that in general the lipids of crenarchaeal viruses were obtained from the host lipid pool, but some lipid species were found to be quantitatively and qualitatively different from the host lipids [50, 53, 54]. Although viral lipids are considered to be derived from the host membrane lipids, it was suggested that they derived from host lipids by modification [53] and possibly by virus encoded enzyme apparatus [50]. Since no such enzyme apparatus has been described in prokaryotic let alone archaeal viruses, the more probable explanation for the differences at the moment is a strong selection for some minor lipid species of the host. Recent study on the assembly of STIV using cryo-electron tomography suggests that the viral membrane is derived *de novo* in the host cell and not as a result of a membrane invagination [31, 71]. This would, at least in theory, allow the possibility that there is viral enzymatic machinery responsible for the lipid modification. The comparative lipid analysis of STIV and its host *S. solfataricus *showed, however, that the viral lipids consisted of a subpopulation of the host lipids but in different proportions [70]. 

### 4.2. Euryarchaeal Viruses

Among euryarchaeal viruses, the icosahedral SH1 and *Haloarcula hispanica *icosahedral virus 2 (HHIV-2) virions contain an inner membrane [40, 42, 72] and the pleomorphic viruses contain a membrane envelope [10, 34, 36, 41]. 

SH1 was the first icosahedral virus characterized among haloarchaea [33]. Inside the rather complex protein capsid, there is a lipid membrane enclosing the approximately 31 kb linear double stranded (ds) DNA genome [28, 40, 42]. The major protein component of the membrane is the approximately 10 kDa VP12, one of the major structural proteins of the SH1 virion [42]. Although there are no detailed studies reporting the assembly steps of SH1, similarities in virion structure to PRD1 suggests a similar assembly pathway [28, 40, 42]. Therefore, it is likely that the viral capsid and the inner membrane are assembled with the help of the membrane proteins and the genome is packaged into these empty particles (procapsid) before the lysis of the cells [28, 33, 40, 42, 73]. Mass spectrometric analysis of the SH1 lipids revealed major archaeal phospholipid species of phosphatidylglycerol (PG), the methyl ester of phosphatidylglycerophosphate (PGP-Me), and phosphatidylglycerol sulfate (PGS). The proportion of PGP-Me, however, was higher in SH1 than in its host *Haloarcula hispanica *[40]. Quantitative dissociation studies of SH1 allowed the separation of the virion into fractions of soluble capsid proteins and lipid core particle (LC) which consisted of the same phospholipid classes and in the same proportions as the intact virions confirming the presence of the inner membrane [42]. Sudan Black B staining was used to show the presence of lipids in the highly purified HHIV-2 virions [72]. Cryo-electron microscopy (cryo-EM) and image reconstruction of SH1 particles show that as in PRD1 [74] and STIV [25] the inner membrane of SH1 follows the shape of the capsid and the membrane is highly curved at the fivefold vertices where there is a clear transmembrane complex probably containing VP2 protein [28]. 

Haloarchaeal pleomorphic viruses is a newly characterized group of viruses with relatively simple virion architecture [10, 34, 36, 41, 56, 73, 75]. The genome (single stranded or double stranded DNA) is enclosed in a membrane vesicle derived from the host membrane [10, 34, 41]. There are two major structural proteins, the larger proteins (approximately 50 kDa in size) are mostly exposed and C-terminally anchored to the membrane [10, 41]. This larger protein is N-glycosylated in HRPV-1 [41, 76], and in HGPV-1 it stains with Sudan Black B [10] suggesting a lipid modification. The smaller structural proteins (approximately 10 to 14.5 kDa) are predicted to contain several trans-membrane domains [10, 34, 41]. New progeny viruses are released from the infected cell without lysis [10, 34, 36]. Thus, the viral envelope is most probably acquired by budding from the sites of host cytoplasmic membrane containing the viral membrane proteins and the genome [10, 34, 36]. The detailed sequence of events and the viral and host proteins involved will be the subject of future studies. Currently, the group of haloarchaeal pleomorphic viruses consists of seven members: *Halorubrum* pleomorphic viruses 1, 2, 3 and 6 (HRPV-1, HRPV-2, HRPV-3, and HRPV-6), respectively [10, 34, 56], *Haloarcula hispanica* pleomorphic virus 1 (HHPV-1) [36], and *Halogeometricum* sp. pleomorphic virus 1 (HGPV-1) [56]. In addition, His2 [55], the second member of genus *Salterprovirus*, is suggested to belong to the pleomorphic viruses [10, 75]. Lipid analysis by TLC or mass spectrometry of the highly purified viral material suggests that the composition of lipids was similar to that of their hosts [10, 34, 41]. The lipids of viruses infecting *Halorubrum *sp. hosts consisted mostly of the archaeal forms of PG, PGP-Me, and PGS, whereas in *Halogeometricum *sp. the PGS was missing both in the host lipids as well as in the lipids of HGPV-1 [10, 41, 77]. Sudan Black B staining of the HGPV-1 and His2 proteins showed that some of the major structural proteins may also be lipid modified [10].

**Figure 2:**
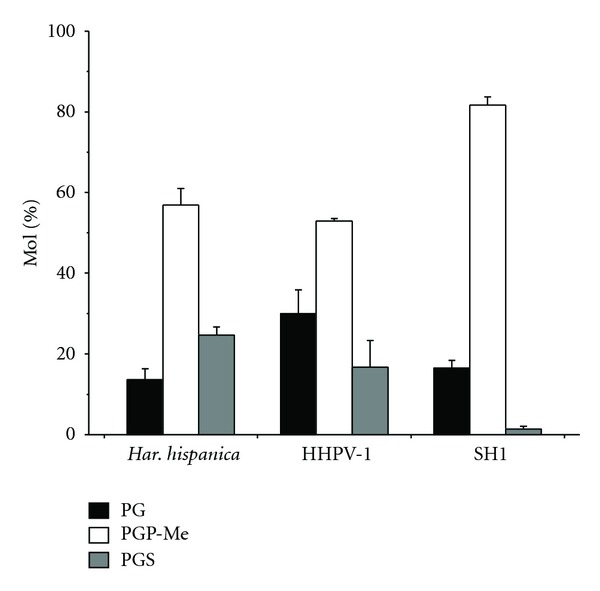
Comparison of the phospholipid compositions of *Har. hispanica*, HHPV-1 and SH1. Concentrations are expressed as the mol% of the total phospholipids. Only phospholipids representing more than 1% of the total are shown. Error bars represent standard deviations of data from at least three independent experiments. Copyright American Society for Microbiology, [36].

Studies on lipid containing haloarchaeal viruses of different morphotypes have also allowed the comparison of the differences in the proportions of incorporated lipids. For example, the isolation and characterization of the *Haloarcula hispanica *pleomorphic virus 1 (HHPV-1) [36] allowed to compare the differences of lipid composition between an icosahedral membrane containing virus SH1and the enveloped, pleomorphic HHPV-1 that infect the same host, *Har. hispanica *(Figure 2). The comparison showed that the lipid composition of the pleomorphic virus HHPV-1 envelope was more similar to the lipids of the host membrane than those of SH1 (Figure 2) [36]. This may be explained by the constraints that the inner membrane curvature poses on the selection of lipids in SH1 and consequently suggests that SH1 is able to selectively acquire lipids from the host membrane [36, 73]. Different lipids are known to have different shapes and therefore can be found in different positions in the curved membrane [14, 78]. It is known that different membrane proteins attract different types of lipids [79], and it would be very interesting to determine which viral proteins are involved in this process.

## 5. Concluding Remarks

Research on lipid containing archaeal viruses is still in its infancy. The presence of lipids and characterization of their nature has been shown for some archaeal viruses [10, 25, 34, 36, 40–42, 50, 51, 53, 54, 58, 59, 70]. Deeper understanding of their role in virus biology is largely still missing. Partly this problem can be assigned to an inability to produce enough material of high enough purity. Partly this problem is due to missing techniques comparable to those developed for lipid research of bacteria and eukaryotes. Lipid research, as many other fields of research, benefits from a thorough characterization of the systems studied. Characterization of the virus life cycle and studying its different steps using the cutting edge technologies in electron microscopy, for example, complements the information obtained using biochemical and genomic methods [25, 28, 31, 40, 42]. The examples set by crystallization of the whole virions of membrane containing bacteriophages PRD1 [74, 80] and PM2 [26] show how valuable different perspectives on lipid membranes can be. Studies on the finding of the proposed viral-encoded genes involved in novel lipid modifications is hampered by the fact that a high amount of predicted gene content in archaeal viruses do not have homologues in the data bases. A more systematic approach of cloning and expression analyses of genes as well as crystallization of the gene products could be used in screening for the functions of interest.

Archaeal lipids are unique in terms of their chemical, physical, structural, and biological properties. Not only can they be admired in their complexity and variability, but as material adjusted to extreme conditions, they can be considered unique for biotechnological applications designed for extreme conditions. The archaeosomes made of one of the major phospholipid of haloarchaeal membranes, the archaeal form of the methyl ester of phophatidylglycerophosphate (PGP-Me), for example, have been shown to be superior in terms of stability and low permeability in high salt conditions [81]. Similar findings were reported for the performance of archaeosomes made of thermophilic lipids in a wide range of temperatures [65]. Although the lipids of archaeal viruses are obtained from host lipids, they can be present in different proportions and the mechanisms for the selection must be driven by viral components. The simplicity of many membrane containing archaeal viruses can be exploited in studying the mechanisms of protein-lipid interplay in archaeal membranes. 

## Figures and Tables

**Table 1:** Currently known membrane containing archaeal viruses, exit strategy, and presence of lipid envelope or inner membrane.

Family or Genus^a^	Type species/example of species/species lipids studied	Exit strategy	Lipids	References
*Globuloviridae *(C)	*Pyrobaculum *spherical virus, PSV	No lysis detected	Lipid envelope	[50]
*Lipothrixviridae *(C)				
Genus *Alphalipothrixvirus *	*Thermoproteus tenax *virus 1, TTV1	Lysis	Lipid envelope	[51, 52]
Genus* Betalipothrixvirus *	*Acidianus *filamentous virus 1, AFV1	No lysis detected	Lipid envelope	[53]
Genus* Gammalipothrixvirus *	*Sulfolobus islandicus *filamentous virus 1, SIFV1	No lysis detected	Lipid envelope	[54]
Genus *Salterprovirus *(E)	His2^b^	No lysis detected	Lipid envelope	[10, 55]

	*Halorubrum *pleomorphic virus 1, HRPV-1	No lysis detected	Lipid envelope	[34, 41]
	*Halorubrum *pleomorphic virus 2, HRPV-2	No lysis detected	Lipid envelope	[10, 56]
*“Pleolipoviridae” *(E)^b^	*Halorubrum *pleomorphic virus 3, HRPV-3	No lysis detected	Lipid envelope	[10, 56]
	*Halorubrum *pleomorphic virus 6, HRPV-6	No lysis detected	Lipid envelope	[10]
	*Haloarcula hispanica *pleomorphic virus 1, HHPV-1	No lysis detected	Lipid envelope	[36]

	*Sulfolobus *turreted icosahedral virus, STIV (C)	Lysis	Inner membrane	[25, 57]
Unclassified	*Sulfolobus *turreted icosahedral virus 2, STIV2 (C)	Lysis	Inner membrane	[58]
	*Sulfolobus tengchongensis *spindle-shaped virus 1 (C)	No lysis detected	Lipid envelope	[59]
	SH1 (E)	Lysis	Inner membrane	[33, 40, 42]

^a^Host domain: B: bacteria, C: crenarchaea, E: euryarchaea.

^b^His2 has been suggested to belong to the new family *Pleolipoviridae. *The approval of the suggested new family is pending at the ICTV.
